# Analysis of fuel pellets from sorted contaminated construction and demolition wood waste

**DOI:** 10.1177/0734242X261429234

**Published:** 2026-03-27

**Authors:** Carina Rehnström, Magnus Ståhl

**Affiliations:** Environmental and Energy Systems, Department of Engineering and Chemical Science, Karlstad University, Karlstad, Sweden

**Keywords:** Wood, pellets, single pellet press, construction, demolition, waste, quality

## Abstract

The construction sector worldwide is producing a massive amount of waste materials. In worst-case scenarios, most of these materials end up in landfills. In the Swedish national waste plan, construction and demolition waste is an area of priority, when transforming from a linear to a circular economy. The aim of this paper is to determine if it is possible to industrially produce good quality pellets from wood waste. The scope includes Swedish construction and demolition wood waste. Four assortments of wood waste from the building sector were turned into fuel pellets in a single pellet press, and their quality was determined. The main pellet quality aspects investigated were moisture content, solid density, hardness, mechanical durability and ash content. The results for these quality aspects were compared to the standards and the results of others. Also, the energy use was determined. The results demonstrate that it is possible to produce good quality pellets from construction and demolition wood waste. The achieved pellet moisture content is such that the investigated wood waste material is suitable for pellet production. The container mix resulted in the highest hardness and durability. However, since the ash content is too high, it should be mixed with pure pine sawdust to make it suitable as a high-quality raw material for the pellet industry.

## Introduction

The Renewable Energy Directive (EU/2023/2413; European Union, 2023) aims at a binding renewable energy target of 45% at EU level by 2030. Within the EU, Sweden has the highest share of renewables in its consumption, approximately 66% (Eurostat, 2023). However, there is a source of renewable energy that is neglected today and that is construction and demolition wood waste.

The construction sector worldwide is producing a massive amount of waste materials. In worst-case scenarios, most of these materials end up in landfills (Hossain et al., 2016) or are incinerated due to lack of working reusing systems. The EU introduced the Construction and Demolition Waste Protocol and Guidelines in 2018 (Directorate-General for Environment, 2018). The Guidelines contribute to the transition to a circular economy and the preparing for reuse, recycling and other material recovery of non-hazardous construction and demolition waste.

According to the Swedish Environmental Protection Agency, 22.6 million tonnes of waste was produced 2022, if mining waste is not included. Of that waste, the construction sector contributes to more than a half, corresponding to about 13.6 million tonnes (Naturvårdsverket, 2024). The construction sector’s waste consists of construction and demolition waste generated during new construction, renovation, remodelling and demolition of buildings. In the national waste plan, construction and demolition waste is an area of priority, when transforming from a linear to a circular economy.

A functioning circular economy with a working reusing system for the wood waste from the construction and demolition sector also decreases the CO_2_-equivalents, from the building sector, and contributes to the global goals on climate actions (Global Goals, 2024). A way to bind CO_2_-equivalents is to reuse building materials. This could be a first step in a consecutive usage of a resource, such as recycled wood, in multiple material functions with energy recovery as the last step (Risse et al., 2019). Risse et al. (2019) did a study on reuse of demolition and construction waste, and one core prerequisite is to preserve the quality of the material in the first steps of the reusing chain. Nowadays, degrading of materials is most common, for example, incineration of solid wood. Instead, solid wood of good quality can be sorted out, for instance by the CaReWood system presented by Irle et al. (2015, 2018) to be used in high-value products like glued and laminated wood products, including finger jointed timber.

Faraca et al. (2019) addressed the challenges with wood waste recycling. Physical and chemical impurities cause problems along the recycling chain. They presented a useful method for sorting wood waste. Their results show that the chemical contamination found in low-quality wood waste is substantially higher than in high-quality wood waste. This indicates that separate collection, classification and management of wood waste can improve the resource quality of wood waste and that it is possible to achieve cleaner recycling methods (Faraca et al., 2019). They also state that 41–87% by weight of the collected wood waste could be recycled, and the rest is impurities. Hossain et al. (2016) also report on contamination of the trace metals Cd, Cr, Cu, Fe, Pd and Zn, but suggest that the waste could go to large-scale energy recovery.

Further, Pettersson et al. (2020) investigated the possibility to recycle ash from wood waste and forest fuels. They conclude that using wood waste materials leads to contaminated wood ash that prevents the recycling of ash, for example to be spread in forests as nutrition to growing trees.

Rautkoski et al. (2016) suggest alternative use of the contaminated construction and demolition wood waste to reach the overall 70% recycling target in EU by 2020. Both mechanical and chemical pulping were tested with rather good results. Wang et al. (2016) also suggest an alternative usage of construction and demolition wood waste: to produce thermal insulating cement-bonded chipboards. They conclude that by optimising the mixture formula, the chipboards produced could meet present standards.

The guidelines ‘Resource and Waste Guidelines at Construction and Demolition’ (Byggföretagen, 2021) aim to improve resource efficiency and waste management in the Swedish construction and demolition sector. The guidelines are a tool to meet the requirements of environmental legislation and to meet society’s expectations for increased circularity with regard to the industries’ materials and waste management. The goal is that material and waste management in construction and demolition projects is carried out in accordance with the waste hierarchy. By sorting the wood waste into different fractions, the waste can be further processed for different purposes. For example, after the impregnated wood is removed, the company PreZero differentiates between clean wood waste and contaminated wood waste (Elofsson, personal communication, 2022). Clean wood waste could be used in new products. The contaminated wood waste goes to incineration or to landfills.

Wood waste from the construction and demolition sector may also become a supply of raw materials for the production of wood fuel pellets, a quality fuel with a high bulk density that makes transportation more effective. In recent years, about 1.8 million tonnes of wood fuel pellets were delivered yearly to the Swedish market, and the raw material is chippings, wood chips and sawdust (Pelletsförbundet, 2024a). In 2023, the wood pellet producers highlighted that there will be a lack of raw materials for pellet production in the years to come (Pelletsförbundet, 2024b). To ensure a stable supply of raw materials, the pellet manufacturers are exploring new non-woody, bio-based residual products, such as agricultural waste.

In Sweden and other Nordic countries, there are some routines and organisations for sorting contaminated construction and demolition wood waste (Byggföretagen, 2021). This is a raw material potential if it is possible to produce pellets from this sorted waste. Since the content of this sort of wood waste differs from country to country, the results cannot be immediately transferred between countries. However, this is not yet investigated in Sweden and other Nordic countries. Internationally, this has been analysed. A study in Costa Rica aimed to evaluate the characteristics of biomass materials from wood waste from the Costa Rican construction sector, its energy potential and its appropriateness for pellet manufacturing. They found that pellets had a calorific value of 19.6 MJ kg^−1^, a solid density of 1.25 g cm^−3^, a bulk density of 720 kg m^−3^, a failure force in compression of 467 N and a durability of 94.28% (Rivera-Tenorio and Moya, 2019). Note that this construction waste contains 10% ash content and that it is contaminated with cement and nails, up to about 6% (Rivera-Tenorio and Moya, 2019). The results obtained for biomass from wood waste are within the range established for pellet quality according to different standards; therefore, wood waste from the Costa Rican construction sector can be used to produce pellets, despite the disadvantage of a high ash content (Rivera-Tenorio and Moya, 2019).

The aim of this work is to determine if it is possible to produce good quality pellets from wood waste. The scope of this paper includes three common fractions of sorted Swedish construction and demolition wood waste. The wood waste pellets are for domestic or industrial use. Four assortments of wood waste from the building sector were turned into fuel pellets, and their quality was determined. The main pellet quality aspects investigated were moisture content, solid density, hardness, mechanical durability and ash content. Also, the energy use was determined. The results for these quality aspects are compared to the standards and the results of others.

## Materials and methods

Three different wood waste materials from construction demolition were pelletised: chipboard, Masonite and Treetex. The fourth pelletised material, the container mix, is a mixture of those three wood waste materials. During pelletising, data were logged to calculate compression work, friction work, maximum force and compressed density. After pelletising, the pellets were measured and tested giving green density, cured density, hardness, mechanical durability, moisture content and ash content.

### Chipboard

Chipboards, also known as particleboards, are made from wood chips and resin. The wood chips, mainly from softwood, are dried and then sprayed with adhesive before pressed together in temperatures up to 200°C to form boards (Dinwoodie, 2001). The adhesive used varies, depending on, for example, location of production. Most chipboards made in Europe formerly used ureaformaldehyde (UF) as adhesive (Dinwoodie, 2001). This is now replaced with aminoplastic adhesives, with very low content of free formaldehyde (Mantanis et al., 2017). When in room temperature, UF emits formaldehyde as gas (allergen and carcinogen; Swedish Chemicals Agency, 2025; Trätek, 1992). Nowadays, the emitted amounts of aldehydes from chipboards have negligible health impacts (Mantanis et al., 2017; Trätek, 1992).

### Masonite

Masonite is a wet-pressed fibreboard made from chipped timber. The wooden chips are steamed under very high pressure that softens the lignin. Thereafter, the chips are put in a defibrator where they are separated into single fibres or fibre bundles. The fibre mass is mixed with hot water and thereafter formed on a wire mesh, cut into lengths and pressed in temperatures ranging from 180°C to 210°C (Dinwoodie, 2001). The density of the product depends on the pressure used during the final pressing. Masonite is a hardboard, and hardboards have densities exceeding 900 kg m^−3^ (Dinwoodie, 2001).

### Treetex

Treetex is a softboard manufactured with the same method as Masonite. The difference is that softboards are pressed with a low pressure in the final pressing resulting in a density less than 400 kg m^−3^ (Dinwoodie, 2001).

### Wood waste sorting

The proportions of the materials included in the container mix are based on a survey of the contents of wood waste containers from construction sites in Sweden. The content of a demolition waste container is sorted into two fractions, that is, waste from structural timber and board materials, representing 56% and 44%, respectively. In Sweden, the majority of the structural timber is made from Scots pine, *Pinus sylvestris*, which is also the main raw material for fuel pellets. The other material fraction, the board material, consists of approximately 30% chipboard, 30% Masonite and 40% Treetex, giving the proportions of the container mix. The study was performed by bachelor students during their diploma work (Toma and Wikström, 2020), under supervision of this paper’s corresponding author.

### Material preparation

The pieces of chipboard, Masonite or Treetex, were sawn into pieces, to suit the capacity of the garden shredder (Stihl GHE 150, Stihl Tirol GmbH, Austria), in which they were shredded before sieving. In a shaking machine from Pascall Engineering (STMN-2-CO402, Crawley, UK), the shavings were sieved during 10 minutes into two fractions (particles less than 5.6 mm and less than 2.0 mm).

The moisture content (wet basis (wb)) was determined according to Swedish Standards Institute (2022b), that is, a portion of 50 g of each material was placed in an oven of 103°C for 24 hours. For the wood waste materials, the particles of the size of 0–2.0 mm were mixed with the particles of 2.0–5.6 mm in the proportion of 50/50. For the chipboard and the Masonite, the two fractions of 50 g each were mixed in a slightly tilted rotating container for 15 minutes. After the mixing, three portions of 30 g each of the two mixed materials were weighed and put in plastic bags. All in all, there were six plastic bags with test samples. The third wood waste material, the Treetex, was very voluminous after the shredding, so the mixture of the two particle sizes had to be done in smaller portions. Hence, portions of 10 g of each fraction size were mixed for 15 minutes. When a total of 100 g had been mixed, the material was divided into three portions of 30 g each and put into plastic bags.

Frodeson et al. (2019a) investigated the moisture effects on density and hardness. Their study showed that the best pellets, produced in a single pellet press, are made with a moisture content (%wb) in a range from 6%wb to 12%wb with an optimum around 8%wb to 10%wb.

The content of the three plastic bags for each mixed material was moisturised to 6%wb, 8%wb and 10%wb, respectively. This was done by adding the needed amount of water to the content of each plastic bag, and blending the water with the shavings in a slightly tilted rotating container for 15 minutes. The moisturised shavings were stored in plastic bags for 24 hours.

### Pellet production

The pellets were produced in the single pellet press described by Frodeson et al. (2018), with the minor modification of excluding the nylon rods (Frodeson et al., 2019b). A sample of 1 g was placed in the steel cylinder of the press, which was set to a temperature of 100°C. The sample was compressed by the piston with the speed of 30 mm min^−1^ to the desired force of 14 kN. A retention time of 10 seconds at full pressure was followed by removal of the bottom plate. The pellet was then pressed out of the cylinder. Previous studies by Frodeson et al. (2018, 2019a, 2019b) act as the base for these settings. For every used material, 12 pellets were made for each of the 3 moisture contents. Directly after falling from the cylinder, each pellet was cooled by a small fan and thereafter placed in a closed plastic bag and stored at room temperature until further analysis.

### Pellet moisture content

The moisture contents of the pelletised materials were determined by analysing the remains from the hardness tests (see below) according to Swedish Standards Institute (2022b). According to Swedish Standards Institute (2021), the moisture content of the pellets *as received* shall be below or equal to 10%wb (M10) for all classes of pellets for commercial and residential applications (A1, A2 and B) as well as for industrial use (I1, I2 and I3). According to the Pellet Handbook, the net calorific value of pellets increases with decreasing moisture content (Obernberger and Thek, 2010). The calorific values of the pellets were not determined in this study. Since this is a feasibility study regarding single pellet production, combustion characteristics are outside the scope. The net calorific values for the wood waste pellets should be above 16.5 MJ kg^−1^ according to Swedish Standards Institute (2021).

### Compression work, friction work and maximum force

During every second of pellet production, the force was logged five times. The product of force and displacement gives the total work, which is comprised of compression work (W_comp_, J) and friction work (W_fric_, J). The time needed to increase the force from 0.5 to 14 kN was the base for calculating the compression work. The pellet moved 10 mm when being pressed out from the cylinder. This displacement, 0–10 mm, is used to calculate the friction work. The maximum force (F_max_, kN) achieved by the piston pressing out the pellet from the cylinder was created by the friction between the surface of the pellet and the die, representing the maximum potential backpressure level created.

### Solid densities

Directly after production, the length, the diameter and the weight of the pellets were digitally measured, giving the solid density of the pellets. After 10 days the pellets were reanalysed. In accordance with earlier studies (Frodeson et al., 2019b; Kaliyan and Morey, 2009; Wongsiriamnuay and Tippayawong, 2015), the newly produced pellets were named ‘green pellets’ and after 10 days ‘cured pellets’. The springback effect on solid densities was determined through variations in axial length and diameter as described by Frodeson et al. (2019b). The reference material, quality Scots pine pellets, with moisture contents of 6%wb to 8%wb, have green and cured densities of 1.25–1.2 g cm^−3^ (Ståhl et al., 2024), that is, the solid density decreases with increasing moisture content.

### Hardness and mechanical durability

The hardness (kg) of the cured pellets were measured using a KAHL motor-driven hardness tester (K3175-0011; KAHL, Reinbek, Germany) described by Frodeson et al. (2018, 2019b). Quality Scots pine pellets, with moisture contents of 6%wb to 10%wb, have a hardness of approximately 20 kg (Ståhl et al., 2024). The mechanical durability, presented as the percentage of pellets, was determined according to Swedish Standards Institute (2015). Both hardness and mechanical durability tests are destructive. Five pellets were tested for mechanical durability according to Frodeson et al. (2019a). For this method, a sample size of five pellets is sufficient. The same number, five other pellets, was used for hardness tests.

The limits for the durability are listed in Swedish Standards Institute (2021). For both commercial and residential applications (classes A1 and A2), as well as for industrial use (class I1), the mechanical durability for pellets must exceed 97.5% (Swedish Standards Institute, 2021). For the lowest class for commercial and residential applications (class B), the corresponding value is 96.5%. The value to exceed for the two lower classes for industrial use, I2 and I3, is 97.0% and 96.5%, respectively. For pellets for industrial use, there is also an upper limit of 99.0% for all three classes.

### Ash content

The ash content (% by weight) of the pelletised materials were determined by analysing the remains from the hardness tests according to Swedish Standards Institute (2022a). For commercial and residential applications, the ash content limits by dry weight are as follows (Swedish Standards Institute, 2021): for class A1 0.7% (A0.7), for class A2 1.2% (A1.2) and for class B 2.0% (A2.0). Corresponding limits for industrial use are for class I1 1.0% (A1.0), for class I2 1.5% (A1.5) and for I3 3.0% (A3.0; Swedish Standards Institute, 2021). According to Savolainen and Berggren (2000), for demolition wood, the ash content can be as high as 37% (Becidan et al., 2012).

## Results and discussion

In order to evaluate if sorted contaminated construction and demolition wood waste could be suitable as pellet raw material, the different calculated and tested quantities are presented and discussed below.

In Table 1, the 12 pellets of each of the 3 moisture content categories for the tested materials are depicted. As seen, most of the produced pellets were of a good or quite good quality. The pellets made of Masonite, Treetex and the container mix were all of a good quality. The pellets made of chipboard of moisture content 6%wb were of a quite good quality, but the pellets of 8%wb and 10%wb were more fragile and some fell apart immediately. To check if this instability of the pellets was due to the size of the shavings, a test series of chipboard with shavings of only one fraction, that is, less than 2.0 mm, was conducted. The result was even worse, since the fragility of the 10%wb series was so high that it was impossible to make pellets.

**Table 1.** Photographs of the 12 pellets of each of the 3 moisture content categories for the tested materials.

Material	Moisture content (wb)		
	6%wb	8%wb	10%wb
Chipboard	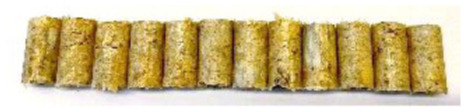	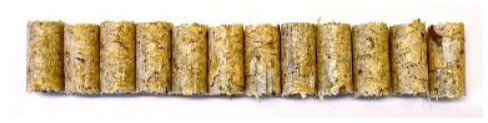	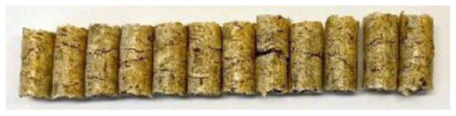
Masonite	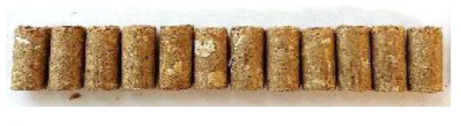	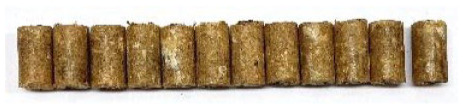	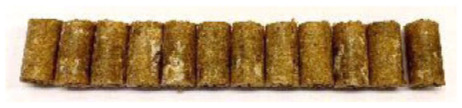
Treetex	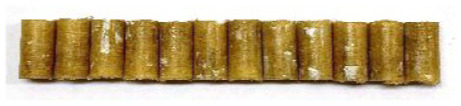	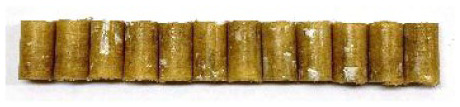	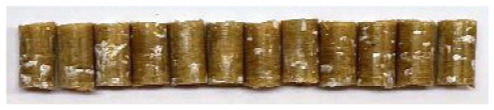
Container mix	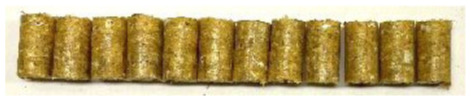	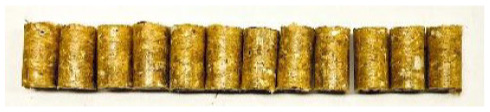	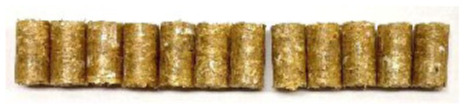

wb: wet basis.

### Pellet moisture content

In Table 2 the denotations of the test series and the moisture contents of the pellets produced are presented. The raw materials were moisturised to result in pellet moisture levels of 6%wb, 8%wb and 10%wb. The objective was to have a 1–2% difference between the test samples, which was achieved for all pellets except T10 and CM10.

**Table 2.** Materials, denotations and moisture contents of the pellets. Also, mean values, based on 12 pellets for each material, for the compression work (W_comp_), the friction work (W_fric_) and the maximum force (F_max_) including standard deviations.

Material	Denotation	MC in pellets (%wb)	W_comp_ (J)		W_fric_ (J)		F_max_ (kN)	
			Mean value	Standard deviation	Mean value	Standard deviation	Mean value	Standard deviation
Chipboard	CB6	7.7	36.2	8.06	1.60	0.30	0.75	0.12
	CB8	8.8	34.1	3.27	1.14	0.19	0.57	0.09
	CB10	9.9	25.6	4.50	1.03	0.18	0.52	0.07
Masonite	M6	6.3	26.1	10.5	3.76	0.54	1.67	0.24
	M8	7.5	23.0	10.9	3.34	0.25	1.79	0.12
	M10	9.5	24.5	9.63	3.37	0.46	1.80	0.18
Treetex	T6	6.8	34.5	8.13	2.51	0.91	1.28	0.44
	T8	8.1	31.2	6.03	1.79	0.43	0.98	0.18
	T10	8.8	24.3	6.43	1.09	0.15	0.54	0.07
Container mix	CM6	5.8	35.7	3.82	1.78	0.15	0.78	0.07
	CM8	7.8	31.6	1.10	1.46	0.14	0.67	0.07
	CM10	8.0	33.6	3.11	1.63	0.18	0.73	0.08

wb: wet basis; MC: moisture content.

A moisture content below 10%wb is achieved for all the pellets produced, which aligns with the target of most fuel pellet industries, and the standard requirements (Swedish Standards Institute, 2021). Generally, lower moisture contents result in higher net calorific values (Obernberger and Thek, 2010). Out of the materials of 6%wb and 10%wb moisture contents, the container mix pellets reach the lowest moisture contents, 5.8%wb and 8.0%wb, respectively, resulting in relatively high net calorific values. For the 8%wb materials, the pellets of Masonite have the lowest moisture content, 7.5%wb, with the container mix as the runner up, with a moisture content of 7.8%wb, resulting in relatively high net calorific values. All in all, the container mix pellets have the highest net calorific values due to the relatively low moisture contents. The moisture contents of pellets from different container mixes may have different characteristics, both positive and negative, resulting in higher or lower net caloric values in the end.

### Compression work, friction work and maximum force

The mean values of the compression work and the friction work are also presented in Table 2. In comparison with the compression work, the friction work seems very low for all of the materials, since they are all below 3.76 J (M6, Table 2). The energy needed to compress the pellets ranges from approximately 23 J (M8, Table 2) to just over 36 J (CB6, Table 2), where both chipboard and Treetex, and their 6%wb and 8%wb series, as well as all the test series for the container mix, have a W_comp_ above 30 J. The rest of the test series have a W_comp_ slightly above 25 J or lower.

The standard deviations for W_comp_ and W_fric_ are also presented in Table 2. For W_comp_, Masonite has the highest differences (M6, M8, M10) from the mean values. The container mix has the lowest (CM6, CM8, CM10). For W_fric_, the highest differences belong to Treetex (T6, T8, T10). The lowest values for W_fric_ are represented by the container mix (CM6) and Masonite (M8, M10). The container mix also has low standard deviations for both CM8 and CM10.

**Figure 1.**
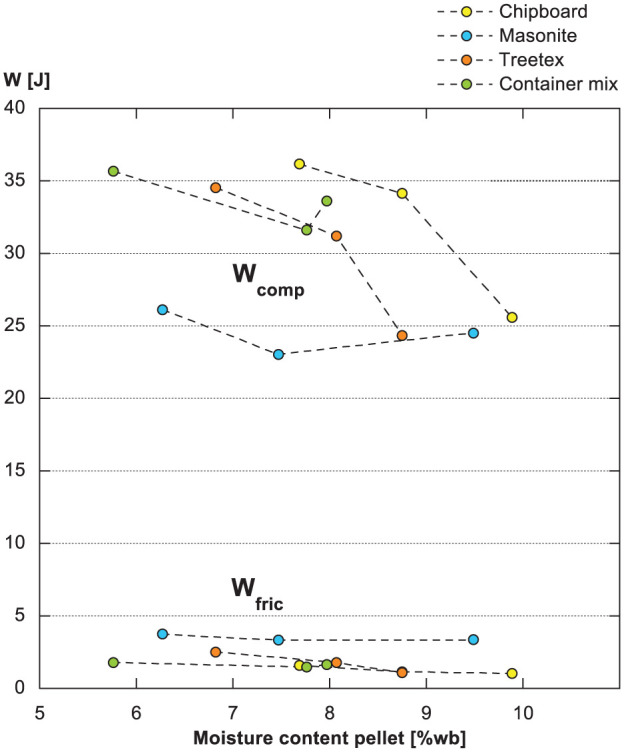
Compression work (W_comp_) and friction work (W_fric_) related to moisture content.

In Figure 1, the compression work and the friction work are related to the moisture contents of the pellets. The W_comp_ values, shown in the upper part of the figure, decrease with an increasing moisture content for chipboard and Treetex. For Masonite, the M6 test series has the highest W_comp_ value and the M9 test series has the lowest. The results for the container mix are the same as for Masonite: the test series with the lowest moisture content results in the highest W_comp_ value and the test series with the intermediate moisture content results in the lowest W_comp_ value. However, all these result tendencies may differ since the standard deviations for W_comp_ are so large. This is indeed the case for Masonite (see Table 2).

In the lower part of the figure, the results for the W_fric_ are shown. The Masonite test series need the highest amount of friction work to produce the pellets. For chipboard, Treetex and the container mix, the W_fric_ values decrease with an increasing moisture content. For Masonite, the 6%wb test series have the highest W_fric_ values and the 8%wb test series have the lowest. The standard deviations for W_fric_ are smaller than those for W_comp_, but the result tendencies may still alter a bit, especially for Treetex for which the standard deviation for the 6%wb series is quite high (see Table 2).

When comparing W_comp_ to W_fric_, the result is that W_comp_ has much higher values than W_fric_, which is also found by Frodeson et al. (2019a). For almost all test series, the W_comp_ and the W_fric_ decrease with increasing moisture content in accordance with Frodeson et al. (2019a). But for the 10%wb series of the Masonite and the container mix, the W_comp_ and W_fric_ increase slightly. This deviation is also seen for M10 and CM10 regarding F_max_.

One indication from the low W_comp_ results of Masonite is that Masonite could have quite low usage of electricity in an industrial pellet press. Another overall indication is that the container mix as well as lower moisture test series of chipboard and Treetex need high inputs of compression work. On the other hand, they are not so demanding when it comes to friction work, in pellet production. This indicates that production of pellets with high moisture contents may be more energy-efficient.

**Figure 2.**
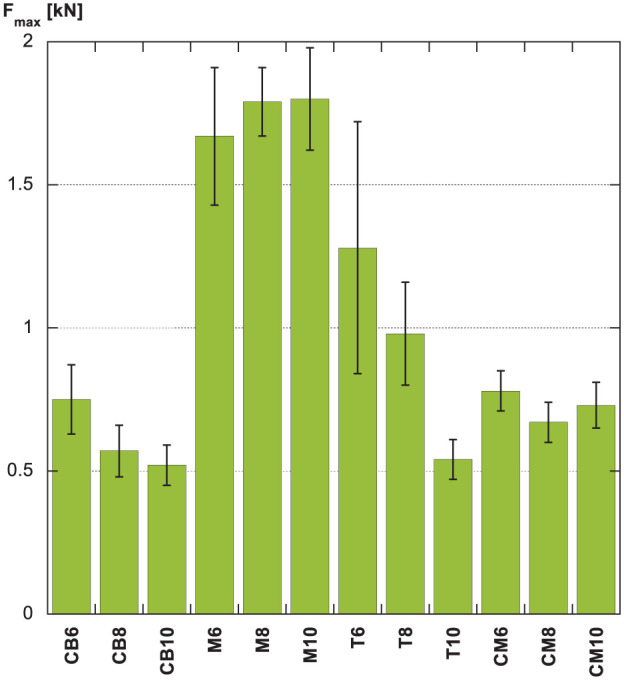
Maximum force (F_max_) for the test series including standard deviations.

The last columns in Table 2 present the mean values and the standard deviations of the maximum force. This is also shown in Figure 2 where it is clearly visible that the highest values for the force were obtained from the test series of Masonite. The chipboard and the container mix series result in quite low maximum forces. The standard deviations for all the test series, except for T6 and T8, are such that they do not affect the overall result. For T6 and T8, the values of F_max_ are relatively high. However, the standard deviations are quite large, so the mean values are not so robust for those test series.

For the maximum force, it is clear that the highest values were obtained from the test series of Masonite. The chipboard and the container mix series result in quite low maximum forces. For pellet manufacturing, the F_max_ result means that Masonite needs more energy to start the transport of the pellets through the die. Likewise, the chipboard and the container mix are more energy-efficient due to quite low maximum forces.

### Solid densities

The pellet solid densities are listed in Table 3. All three container mix series (CM6, CM8, CM10) as well as two of the Treetex series (T8, T10) are clearly the highest when it comes to the compressed solid density. For those test series, the compressed densities are all above 1.4 g cm^−3^, with the highest slightly above 1.6 g cm^−3^ (1.44–1.61 g cm^−3^). For the rest of the test series, the compressed densities range from 1.24 g cm^−3^ (M6) to 1.35 g cm^−3^ (T6). One test series has green and cured densities below 1.0 g cm^−3^, that is, CB10 (0.95, 0.97 g cm^−3^). The pellets of this test series were more fragile and some fell apart directly. The rest of the test series have green and cured densities ranging from 1.07 g cm^−3^ (CB8, green and cured) to 1.29 g cm^−3^ (T6 and T10, both cured). All of the values for the pellet solid density are very robust since their standard deviations are small. Overall, the density decreases from compressed to green solid density, but the 10 days of rest, to achieve cured solid density, have no effect.

**Table 3.** Solid density for compressed, green and cured pellets, where the values are from all 12 pellets in each moisture content set. Also, data for hardness and mechanical durability, where the values are based on five pellets for each test series. Durability values marked with dashes were lower than 90%. When test values go below 90%, the tests, and thereby measured values, are not reliable.

Material denotation	Pellet solid density (g cm^−3^)						Hardness (kg)		Durability (%)
	Compressed		Green		Cured				
	Mean value	Standard deviation	Mean value	Standard deviation	Mean value	Standard deviation	Mean value	Standard deviation	
CB6	1.29	0.01	1.12	0.01	1.14	0.02	5.2	0.4	—
CB8	1.31	0.01	1.07	0.02	1.07	0.02	5.6	2.2	—
CB10	1.30	0.01	0.95	0.02	0.97	0.02	2.5	0.5	—
M6	1.24	0.02	1.14	0.02	1.14	0.02	7.0	1.8	—
M8	1.28	0.01	1.17	0.01	1.17	0.01	8.3	1.9	95.8
M10	1.30	0.01	1.11	0.02	1.12	0.01	6.4	1.1	100.0
T6	1.35	0.01	1.28	0.01	1.29	0.02	10.3	0.4	100.0
T8	1.44	0.02	1.26	0.02	1.28	0.02	11.4	0.7	100.0
T10	1.49	0.02	1.27	0.01	1.29	0.01	11.2	0.6	100.0
CM6	1.53	0.02	1.20	0.02	1.21	0.01	23.8	0.3	100.0
CM8	1.56	0.03	1.18	0.02	1.19	0.01	24.0	0.0	100.0
CM10	1.61	0.03	1.21	0.03	1.22	0.02	23.9	0.2	98.0

**Figure 3.**
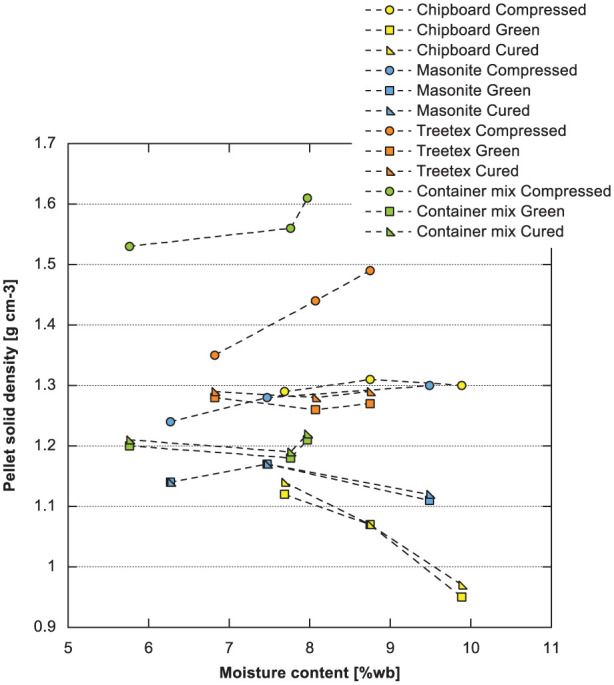
Solid density for compressed, green and cured pellets related to moisture content.

In Figure 3, the pellet solid density is related to the pellet moisture content. The compressed density increases with increasing moisture content for the Masonite, the Treetex and the container mix series. The compressed density for the chipboard seems not affected by the moisture content. The green and cured density values for the Treetex and the container mix series are not affected by the increasing moisture content. For the Masonite series, the densities are affected a bit more, with the lowest densities for the highest moisture contents. For the chipboard series, it is clear that the green and cured densities decrease with increasing moisture content.

Both the chipboard and the Masonite series have green and cured pellet solid densities lower than that of quality Scots pine pellets (Ståhl et al., 2024). However, the Treetex and the container mix series reach the requested green and cured solid density of 1.2–1.25 g cm^−3^, with the Treetex series’ densities even above the higher value.

For all the materials, the solid density for compressed pellets increases with increasing moisture content, except for the chipboard series for which the 10%wb series’ solid density is lower than that for the 8%wb series. All solid densities decrease from compressed to green pellets, but 10 days of storage gives no further change in solid density. The same springback effect relations between a relatively high compressed density and more equal green and cured densities were also found by Berghel et al. (2022) and Frodeson et al. (2019b). Note that the different materials tested have different length of fibres, which affects the expansion properties of the material.

### Hardness and mechanical durability

As shown in Table 3, the hardness of the container mix pellets is outstanding compared to the other materials. Also, the container mix results have very small standard deviations meaning that the results are very robust. The hardness for the container mix is around 24 kg, whereas the other materials’ hardness ranges from 2.4 to 11.4 kg, that is, a maximum of less than half that of the container mix for T8 at 11.4 kg. In addition, most of the standard deviations, except for Treetex, for the other materials are much larger.

The pellet hardness of the container mix series is in the range of the hardness of quality Scots pine pellets according to Ståhl et al. (2024). Therefore, the container mix is well suited as an industrially produced pellet raw material. The hardness of the three other tested materials is well below that of the container mix. For all test series, the hardness seems unaffected by moisture content except for CB10 and M10. The low hardness for these two test series may be due their relatively low W_comp_. However, the standard deviations for both the CB-series and the M-series are quite large; hence a conclusion is hard to draw.

The mechanical durability exceeds the lower limit, 97.5% for first class pellets, for Masonite with 10%wb moisture content and for all of the Treetex and the container mix test series. All the other test series have too low values to be classified. In combination with the upper limit for class I1, the container mix with a moisture content of 8%wb is the only test series that is within limits. For industrial use, this means that Treetex, or mixes with Treetex, is most suitable for pellet production. Hence, the result of the material series with too low a durability would become better by adding Treetex.

For industrial production, it is important that the durability of the wood waste pellets exceed the requirements of the Swedish standards. This improves the outcome from the production, reduces the number of fines during transportation and facilitates for consumers.

### Ash content

The ash contents for the tested materials are listed in Table 4. As shown, Masonite, with an ash content of 1.5%, is classified in the lower classes B and I2 with the upper limits of 2.0% and 1.5%, respectively. The other materials, Chipboard (3.5%), Treetex (9.6%) and the container mix (5.4%), have too high ash contents to be classified.

**Table 4.** Ash content for the different materials in percentage by dry weight. The two final columns show which criteria the different materials fulfil according to the standard (Swedish Standards Institute, 2021).

Material	Ash content (%)	Commercial and residential applications	Industrial use
Chipboard	3.5	—	—
Masonite	1.5	B (A2.0)	I2 (A1.5)
Treetex	9.6	—	—
Container mix	5.4	—	—

As shown in Table 4, Masonite, with an ash content of 1.5%, is classified in the lower classes B and I2 with the upper limits of 2.0% and 1.5%, respectively. The other materials, Chipboard, Treetex and the container mix, have too high ash contents to be classified. Hence, when mixing assortments of construction and demolition wood waste, the portions should be such that the upper ash content limits are not exceeded to fulfil the standard requirements.

### Discussion summary

In summary, for the pellet industry it is important that the right moisture content is possible to achieve. This is achieved for all tested materials. Further, the compression work and the friction work must be high enough to produce durable high-quality pellets. This is the case for the Treetex and the container mix. A low ash content is also important for high-class standard pellets. The only material with low enough ash content to be classified is Masonite.

The content of the container mix used in the test series is based on three fractions of board materials in a sorted construction and demolition wood waste container. Another fraction, waste from structural timber, may have a very high ash content (Becidan et al., 2012) and cannot then be used to reduce the ash content of pellets. Instead, the content from a demolition wood waste container may be mixed with pure pine saw dust to fulfil the standard requirements for ash content.

## Conclusions

In this paper, it is shown that it is possible to produce good quality pellets of Swedish construction and demolition wood waste. This is the first such study in the Nordic context. Four assortments of wood waste from the building sector were turned into fuel pellets, and their quality was determined.

1. For the quality criteria compression work and friction work, a not-so-robust conclusion is that production of pellets with high moisture contents may be more energy-efficient.
2. For the quality criteria maximum force, a robust conclusion is that for pellet manufacturing, Masonite needs more energy to start the transport of the pellets through the die. Likewise, another robust conclusion is that the chipboard and the container mix are more energy-efficient due to quite low maximum forces.
3. For the quality criteria solid density, a robust conclusion is that for all materials, except for the chipboard series with a 10%wb moisture content, the solid density for compressed pellets increases with increasing moisture content. Another robust conclusion is that all solid densities decrease from compressed to green pellets, but 10 days of storage gives no further change in solid density.
4. For the quality criteria hardness, a robust conclusion is that the container mix is well suited as a raw material for industrial pellet production.
5. For the quality criteria mechanical durability, a robust conclusion is that Treetex, or mixes with Treetex such as the container mix, is most suitable as a raw material for industrial pellet production.
6. For the quality criteria ash content, a conclusion is that, except for Masonite, the investigated waste materials need to be mixed with low ash content materials such as pure pine sawdust to fulfil the standard requirements.

The recommendations for further work are

- To investigate other construction and demolition wood waste mixes.
- To investigate the use of additives to improve the pellet qualities.
- To investigate the effect on other quality criteria when mixing with pure pine sawdust to reduce the ash content.

When such studies result in high-quality pellets, the investigation should also be extended to include net calorific value of such pellets.
